# Creation of a rich vascular subcutaneous space for cell transplantation via injectable biological hydrogels

**DOI:** 10.1038/s41598-025-29873-8

**Published:** 2025-12-20

**Authors:** Asmaa Samy, Ghada A. El-Sherbeny, Sherry M. Khater, Haytham G. Aamer, Mohamed A. Abdelhameed, Ayman F. Refaie

**Affiliations:** 1 Biotechnology Department, Urology and Nephrology Center, Mansoura University, Mansoura, Egypt; 2 Botany Department, Faculty of Science, Mansoura University, Mansoura, Egypt; 3 Pathology Department, Urology and Nephrology Center, Mansoura University, Mansoura, Egypt; 4 Animal Research Department, Urology and Nephrology Center, Mansoura University, Mansoura, Egypt; 5 Nephrology Department, Urology and Nephrology Center, Mansoura University, Mansoura, Egypt

**Keywords:** Neovascularization, Subcutaneous site, Cell transplantation, Biocompatible hydrogels, Biotechnology, Materials science, Medical research, Stem cells

## Abstract

The subcutaneous space offers an attractive and accessible site for cell transplantation. However, its clinical utility is often hindered by insufficient vascularization. This study evaluated the vascularization potential of three hydrogels; human collagen type I, human fibrin, and alginate implanted subcutaneously in Sprague-Dawley rats. Polylactide-co-glycolide (PLG) scaffolds used as positive controls for foreign body response. Sequential injections were performed at 1, 2, and 4 weeks, and tissues were retrieved for histopathological examination. Human collagen type I and fibrin induced robust neovascularization compared to controls, with peak vessel density at week 1 (2.79-fold and 3.18-fold; *P* < 0.001) and sustained increases through week 4 (1.94-fold and 2.3-fold, respectively). No significant differences were observed between human collagen type I and fibrin at any time point. Both biomaterials were well tolerated without evident of fibrosis or foreign body reaction. Alginate produced the strongest early angiogenic effect (3.96-fold at week 1; *P* < 0.001) but was associated with marked inflammation and fibrosis by week 4. PLG scaffolds induced modest vascularization but consistently provoked inflammatory reactions and fibrosis. Human-derived hydrogels thus combine rapid and durable vascularization with excellent biocompatibility, providing a minimally invasive strategy to enhance subcutaneous cell transplantation outcomes.

**Supplementary Information:**

The online version contains supplementary material available at 10.1038/s41598-025-29873-8.

## Introduction

Organ transplantation is considered the gold standard therapeutic strategy for end stage organ failure. However, the persistent global donor organ shortage, together with complex ethical dilemmas^1^, emphasizes the need to investigate alternative modalities. With the significant developments in recent years, cell transplantation is considered a promising option^2^. In alignment with our experimental research aiming for treatment of diabetes mellitus by stem cell transplantation, we were in bad need for suitable implantation site. Over the past 17 years, in a consecutive series of experiments, our group was able to generate functional insulin-producing cells (IPCs) derived from human mesenchymal stem cells (MSCs)^3–14^. However, successful cell transplantation is still hindered by several obstacles. One of the major challenges is the selection of an optimal implantation site. In this context, numerous anatomical sites have been investigated; the renal subcapsular space^4,5,7,15–18^, the subcutaneous tissue^17–21^, lymph nodes, spleen, intraperitoneal cavity, muscle compartments, and the intravenous vasculature^20,22,23^.

The subcutaneous site offers several advantages, including easy accessibility and capacity for accommodating substantial cell populations. Furthermore, the procedural amenability to a minimally invasive approach supports its clinical feasibility. However, one of the inherent limitations of this site is its meager vascularity impeding the angiogenic processes which is critical for sustaining cell viability and promoting cellular function^17,19–21,23^. To overcome this limitation, several complicated strategies have been proposed. The natural structural design of decellularized extracellular matrix scaffolds makes them suitable for a vascularization environment^24–26^. The advancement of in situ prevascularization adopting the flap technique that uses a vascularized tissue flap embedding for a construct before transplantation into the target site. This method was successfully applied in mandibular reconstruction with prevascularized scaffolds in clinical practice^25,27^. Another sophisticated method employs the arteriovenous loop model to construct a vascularized bed through surgical anastomosis of the artery and vein within a protected chamber containing a scaffold^25,28–30^. Hydrogel materials like fibrin and Matrigel were used in these chambers to facilitate vessel sprouting and maturation^25,31^. Synthetic scaffolds like polylactic-co-glycolic acid (PLGA) and processed bovine cancellous bone (PBCB) were also used in growth chambers to enhance vascular network formation^25,32^.

Vascular endothelial growth factor (VEGF) and basic fibroblast growth factor (bFGF) play a vital role in enhancing blood vessel formation and maturation^25,33^. Yet, dysregulation of VEGF dynamics has been implicated in various pathological states, encompassing neoplastic disorders and retinal degenerative afflictions such as diabetic retinopathy and age-related macular degeneration^34^. Despite the promising prospects of VEGF for therapeutic angiogenesis, the translation of such advantages into clinical progress has been impeded by the intrinsic limitations of VEGF protein administration. This has been attributed to its abbreviated half-life and poor tissue retention upon direct injection^35^.

Alternatively, biomaterial hydrogels namely; human collagen type I, human fibrin, and alginate could offer a promising strategy for creation of an efficient vascular environment in the subcutaneous space. Collagen type I is a crucial biomaterial in cardiac and vascular graft applications. It is the main component of vertebrate connective tissues and affects tissue architecture and mechanical integrity^36,37^. Fibrin, derived from the fibrous meshwork of blood clots, offers inherent angiogenic properties, rendering it an attractive choice for tissue engineering and vascular regeneration^36^. Alginate, a natural biocompatible and biodegradable polysaccharide, provides the ability for the fabrication of different scaffold forms, including microspheres, microcapsules, and fibrous structures^38^.

Therefore, the present study aimed to investigate the potential impact of three biocompatible biological hydrogels namely; collagen type I, fibrin, and alginate in promoting host-driven neovascularization in the subcutaneous tissue of Sprague Dawley (SD) rats. The main objective was to establish a pre-vascularized site suitable for future cell transplantation.

## Materials and methods

The aim of this study was to compare the neovascularization potential and tissue response induced by three injectable hydrogels: human collagen type I, human fibrin, and alginate in the subcutaneous space of SD rats. PLG scaffold group as a positive control for foreign body reactions. This experimental, controlled animal study was conducted at the animal facility of the Urology and Nephrology Center, Mansoura University, Egypt. The required approval for this study was obtained from the Animal Care and Use Committee of the University of Mansoura (MU-ACUC: SC.MS.23.11.49). All methods were carried out in accordance with relevant guidelines and regulations. All methods are reported in accordance with ARRIVE guidelines (https://arriveguidelines.org).

### Animal model and housing conditions

Twenty-seven female SD rats, weighing 250–280 g each, were kept in polycarbonate cages under a controlled environment of light and dark cycle at a temperature of 24 ± 3 °C and relative humidity of 50–60%. Room lighting was automatically turned on and off at 7 AM and 7 PM. All rats were subjected to a ten-day acclimatization period prior to the procedures.

### Chemicals and kits

Collagen type I solution (3 mg/mL), derived from human fibroblasts, was obtained from Sigma-Aldrich (St. Louis, MO, USA). The fibrin kit, containing human fibrinogen (6.5 g/dL) and human thrombin (1000 IU/mL), was purchased from Fibrogloo Fibrin Sealant (Power of Platelets, Singapore). Sodium alginate powder was obtained from Alpha Chemika (Mumbai, India), and polylactide-co-glycolide (PLG) scaffolds were purchased from Advanced BioMatrix (Catalog No. 5239-1EA, Carlsbad, CA, USA). For histopathological examination, normal swine serum (Dako, Glostrup, Denmark), CD34 antibody (Catalog No. ab81289, Abcam, Cambridge, UK), and a biotinylated secondary antibody (StreptABComplex/HRP Duet, Dako, USA) were used. Gill’s Hematoxylin was obtained from Vector Laboratories (Peterborough, UK). A fluorescently labeled secondary antibody (Alexa Fluor 488-conjugated anti-mouse IgG, #4408, Cell Signaling Technology) and the nuclear counterstain 4′,6-diamidino-2-phenylindole (DAPI; #4083, Cell Signaling Technology) were also used for immunofluorescence imaging.

### Preparation of hydrogels and scaffold

#### Collagen type I hydrogel:

Human collagen type I was provided in a sterile liquid form at a concentration of 3 mg/mL which is converted to gel state at 37 °C after injection.

#### Fibrin hydrogel:

Lyophilized sterile human fibrinogen (6.5 g/dL) and human thrombin (1000 IU/mL) were reconstituted by dissolving each vial in 2 mL of water for injection to form a hydrogel instead of a clot, resulting in a final concentration of 3.25 g/dL for fibrinogen and 500 IU/mL for thrombin.

#### Alginate hydrogel:

A 5% (w/v) sodium alginate hydrogel was prepared by dissolving the powder in warm distilled water with continuous stirring, followed by sterilization using an autoclave at 121 °C for 15 min.

#### polylactide-co-glycolide (PLG) scaffold:

A 4.5 × 4.5 × 0.15 cm PLG sheet was sectioned into small squares measuring 0.5 × 0.5 cm using a sterile scalpel. Each piece had a surface area of 0.25 cm² and a uniform thickness of 0.15 cm. The cut pieces were then sterilized using low-temperature plasma sterilization. From these, nine identical pieces were selected for subcutaneous implantation.

### Animal preparation

A total of 24 SD rats were divided into three groups: Human collagen type I, Human fibrin, and Alginate (8 rats each). A fourth group of 3 rats were implanted with PLG scaffolds served as a positive control group for foreign body reactions.

Anesthesia was induced in two stages. First, inhalation was applied using isoflurane, followed by intraperitoneal injection with xylazine (10 mg/kg) and ketamine (80 mg/kg). Once anesthetized, the dorsal surface of each rat was shaved and then sterilized using alcohol and betadine.

### Hydrogel injection, scaffold implantation, and tissue collection

To standardize the injection sites, the dorsal skin was divided into four equal quarters using a sterile steel ruler. Each rat received subcutaneous injections of the same hydrogel at the three designated dorsal sites at different time points (1, 2, and 4 weeks). The fourth quadrant was spared from injection and served as a self-normal control. Similarly, PLG scaffolds were implanted subcutaneously at the designated three sites following the same dorsal quadrant-based distribution and time-point tracking.

#### Collagen type I hydrogel:

Under sterile conditions, a volume of 300 µL of the liquid human collagen type I was injected subcutaneously.

#### Fibrin hydrogel:

A total of 250 µL of fibrinogen and 250 µL of thrombin were separately loaded into two separate insulin syringes. The two solutions were injected and mixed at the same time by using a tri-Luer lock for the application of the dual injection technique.

#### Alginate hydrogel:

A total of 300 µL of the prepared solution was administered via subcutaneous injection with an insulin syringe.

Each rat received the three injections of the same hydrogel at separate dorsal sites. Injections were administered sequentially at weekly intervals, starting with the site designated for the 4-week endpoint, followed by the 2-week and finally the 1-week sites. This allowed longitudinal evaluation of hydrogel effects within the same animal. Regularly, the quarters were remarked daily to ensure that the injection sites remained clearly identified throughout the study period.

#### PLG scaffolds:

Each rat received 3 pieces of the scaffold in the three designed sites. Each piece was implanted subcutaneously through a 1 cm skin incision. Following implantation, the incision was closed using 4 − 0 coated VICRYL sutures. All procedures were carried out under complete aseptic conditions.

Following each injection or implantation, the injected site was re-sterilized using betadine.

At the end of the study, the rats were euthanized using overdose of isoflurane inhalation. The subcutaneous tissues of the marked quarters were excised for histopathological examination to assess the effect of each hydrogel.

### Macroscopic evaluation

The injected subcutaneous tissues were macroscopically evaluated for signs of neovascularization, characterized by increased localized redness and visible blood vessel networks. Photographic documentation was performed to compare the vascular responses among all groups at various time points.

**Histopathological examination**

A) **Assessment of neovascularization**

The preliminary assessment of the newly formed blood vessel was evaluated by Hematoxylin and Eosin (H&E). For visualization and quantification of blood vessels, formalin-fixed paraffin-embedded sections from each specimen were stained with anti-CD34 antibody. Tissues were stained as follows; briefly, 4-µm sections were deparaffinized in xylene, followed by rehydration in ethanol. Antigen retrieval was performed in 0.01 mol/L sodium citrate buffer (pH = 6) in a microwave. Endogenous peroxidase activity was blocked in 0.01% hydrogen peroxide in methanol, and non-specific binding was blocked using normal swine serum. The primary antibody (CD34, 1:150) was incubated with the tissue for 1 h before incubation with a biotinylated secondary antibody according to the manufacturer’s instructions. CD34 reaction was accomplished with 3,3′ diaminobenzidine (DAB) as the chromogenic peroxidase substrate. Sections were counterstained with Gill’s Hematoxylin formula, dehydrated, and fixed before mounting with DPX. Sections of placental tissue were included as positive controls, where the primary antibody was omitted for negative controls.

Vascular density was assessed by counting all positively stained blood vessels in the whole section. Briefly, the sections were divided into ten areas; positively stained vessels were counted across the total subcutaneous tissue area. Vessel density in each area was determined by counting the sum of small vessels in that area using a transmission light microscopy (Olympus, BX51, Tokyo, Japan) and ImageJ analysis software (developed by NIH). The median was estimated for the 10 fields.

In addition to chromogenic detection, selected sections, obtained from the same formalin-fixed, paraffin-embedded (FFPE) tissue blocks, were also subjected to immunofluorescence (IF) staining to enhance visualization of the vascular architecture. Following deparaffinization and rehydration, antigen retrieval was performed using sodium citrate buffer (0.01 mol/L, pH 6.0) in a microwave, as described for immunohistochemistry (IHC). To block non-specific binding, sections were incubated with 5% normal goat serum for 1 h at room temperature. Slides were then incubated overnight at 4 °C with a primary anti-CD34 antibody (1:100). After washing, sections were incubated for 2 h in the dark with a fluorescently labeled secondary antibody according to the manufacturer’s instructions. Nuclear counterstaining was performed using 4′,6-diamidino-2-phenylindole (DAPI). Finally, sections were mounted using an aqueous anti-fade medium and stored in the dark until imaging. Confocal digital images were captured using a Leica TCS SP8 microscope (Leica Microsystems, Mannheim, Germany).

B) **Assessment of inflammatory and foreign body tissue reactions**

Sections from the injected subcutaneous tissue were stained with H&E to determine the extent of any inflammatory or foreign body tissue reactions. For fibrosis, sections were stained blue with Masson’s trichrome.

### Statistical analysis

Statistical analyses were performed using SPSS for Windows, version 16.0. non-parametric statistics were used in this study. Therefore, the median was used to represent the data in which no deviation or error bars existed. For changes over time in each group, a Friedman test was used. For testing four non- parametric independent samples, the Kruskal-Wallis H test was used for measuring differences, while Mann-Whitney test was used to detect the significance in between each two groups, for groups which were significant in Kruskal-Wallis H test. A *p-value* of ˂ 0.05 was considered statistically significant.

### Result

#### Macroscopic evaluation for vascularization and tissue reaction

**Fig. 1:**
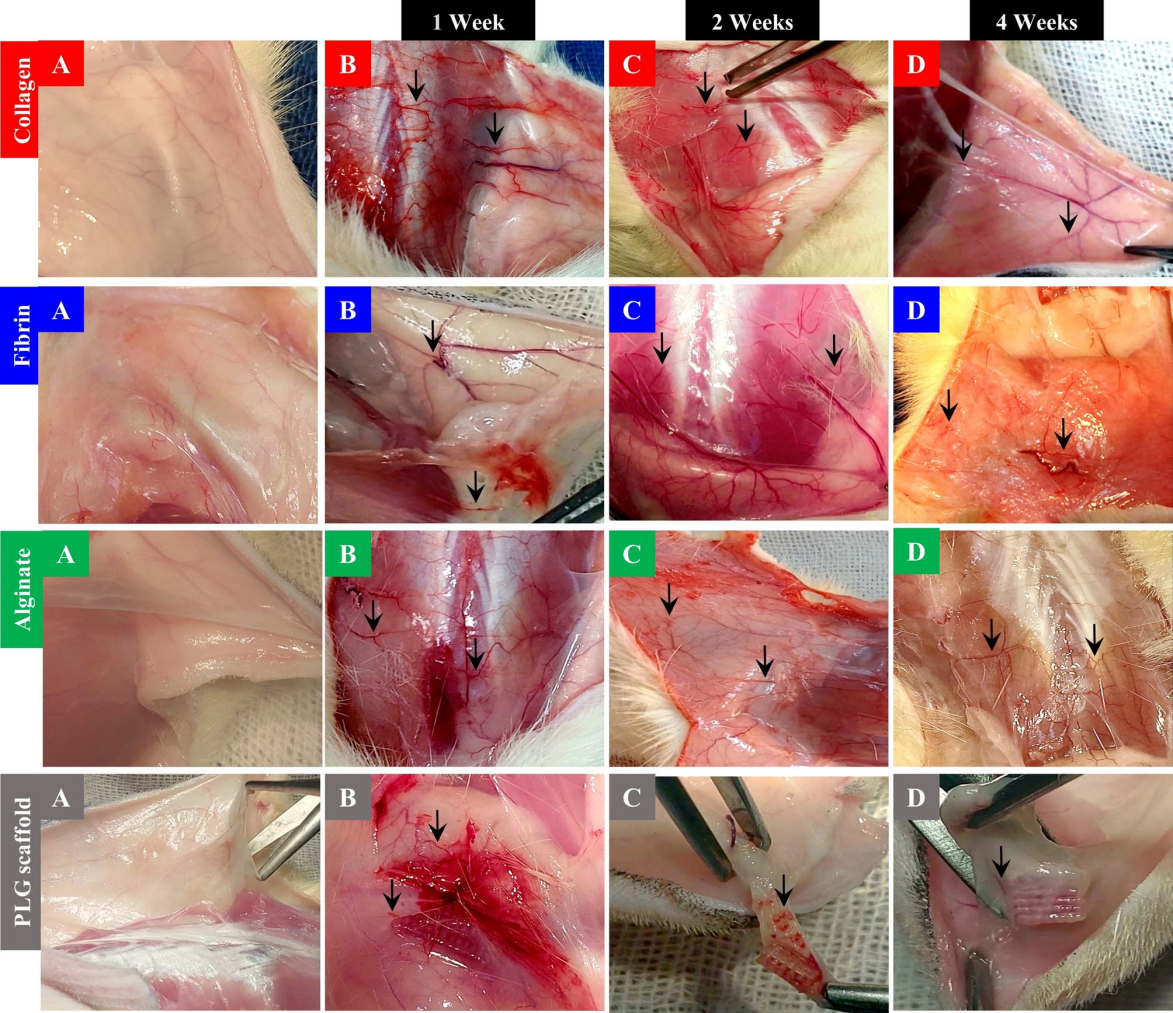
Macroscopic images of the subcutaneous tissues in all groups at all time points: **A**: Normal control site displaying intact tissue architecture without notable neovascularization or inflammation. **B.** One-week post-injection; human collagen type I and fibrin groups showed evidence of early neovascularization with mild redness consistent with increased microvessels formation with no visible foreign body reaction. The alginate and PLG scaffold groups demonstrated inflammatory swelling and localized redness. **C.** Two weeks post-injection; human collagen type I and fibrin groups maintained visibly increased vascularity without fibrosis. However, the vascular density slightly decreased compared to the 1-week timepoint. Alginate and PLG scaffold groups displayed sustained irregular redness. **D.** Four weeks post-injection; human collagen type I and fibrin groups retained visible vascular structures indicative of sustained neovascularization without external signs of fibrosis. Although the vascular density was lower than the 1-week time point, it was still higher than normal tissue. The alginate and PLG groups exhibited fibrotic reactions with reduced vascularity. Black arrows indicate newly formed blood vessels.

In human collagen type I and fibrin groups, the injected subcutaneous tissues exhibited early and sustained signs of increased blood vessel formation, in the form of macroscopically evident hyperemia indicative of neovascularization without evident fibrosis. This neovascularization was apparent from the first week and remained persistently visible at the two and four weeks timepoints. In contrast, alginate and PLG scaffold groups showed initial redness associated with inflammation, which progressed to fibrous tissue formation by week four. Despite some vascular features, the persistent abnormal redness in these groups were indicative of inflammatory reactions rather than functional neovascularization (Fig. 1).

### Microscopic evaluation of neovascularization

#### Hematoxylin and Eosin (H&E) staining

**Fig. 2:**
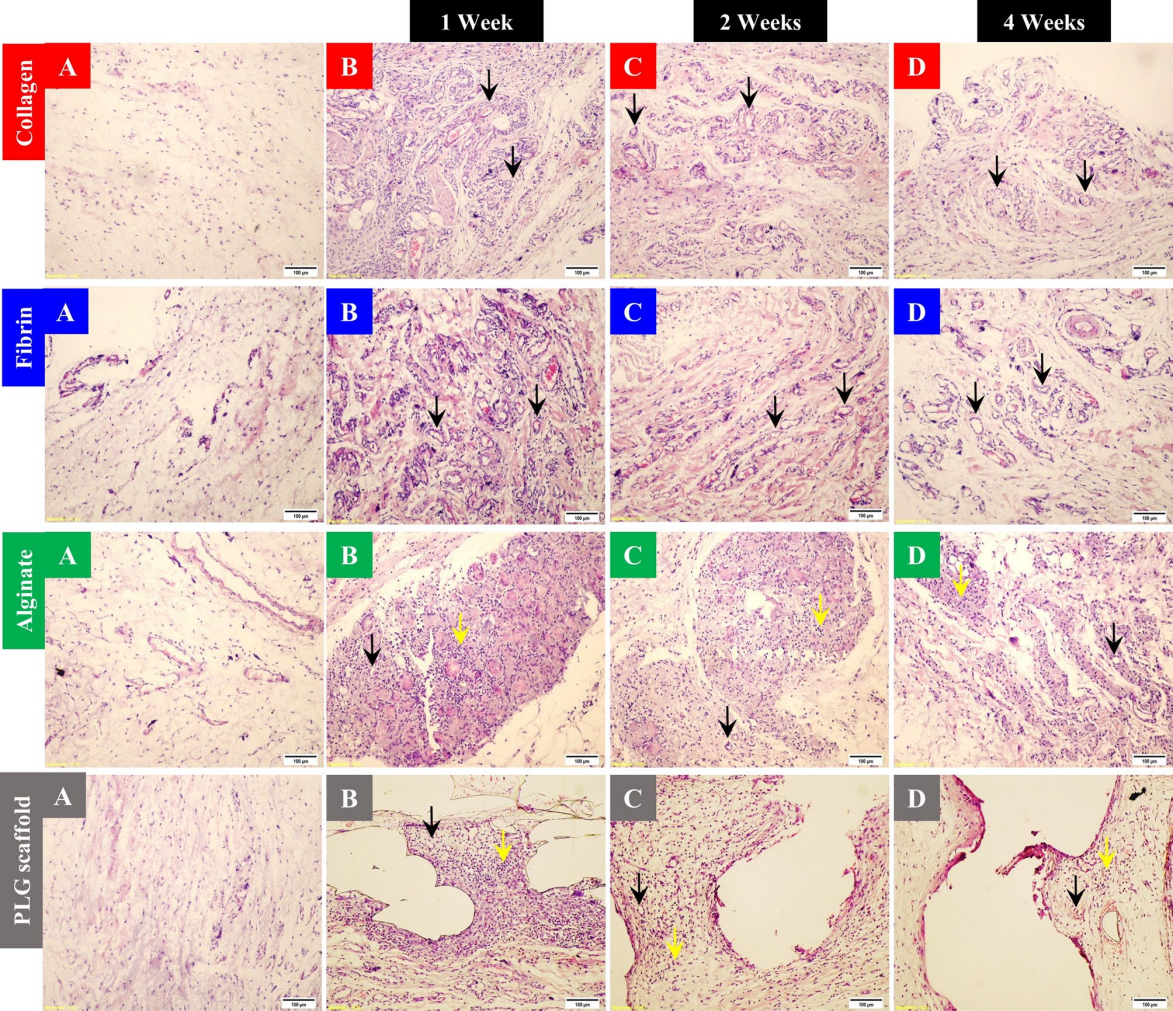
Histopathological examination of the subcutaneous tissues in all groups (H&E; × 10 magnification): **A**: Normal control tissue exhibiting intact adipose architecture. **B**: One-week post-injection; both human collagen type I and fibrin groups displayed increased microvessels formation compared to control, with no foreign body reaction. The alginate and PLG scaffold groups showed blood vessel formation with inflammatory cell infiltration and fibrotic tissue deposition. **C**: Two weeks post-injection; vascular density remained increased in human collagen type I and fibrin groups without stimulation of inflammatory response. Alginate and PLG scaffold samples showed persistent inflammation with elevated vascular density. **D**: Four weeks post-injection; human collagen type I and fibrin showed the increased vascular density. There was no inflammation provoked by both hydrogels. Alginate and PLG scaffold groups maintained the increased vascular density and increased inflammatory reactions and fibrotic responses throughout. Black arrows indicate blood vessels, and yellow arrows indicate inflammatory cell infiltrates. Scale bar = 100 μm.

H&E staining in the human collagen type I and fibrin groups revealed well-developed mature adipose tissue across all samples. The treated tissue architecture remained intact, and newly formed blood vessels exhibited a leaky morphology. A marked increase in the number of blood vessels was observed compared to the normal control tissues. In the alginate group, H&E staining revealed neovascularization accompanied by dense inflammatory infiltrates, which veiled early morphological assessment. Similarly, the PLG scaffold group elicited a vascular response comparable to the alginate group, with identifiable microvessels and persistent inflammation **(**Fig. 2**)**.

### Immunohistochemistry (IHC) staining

**Fig. 3:**
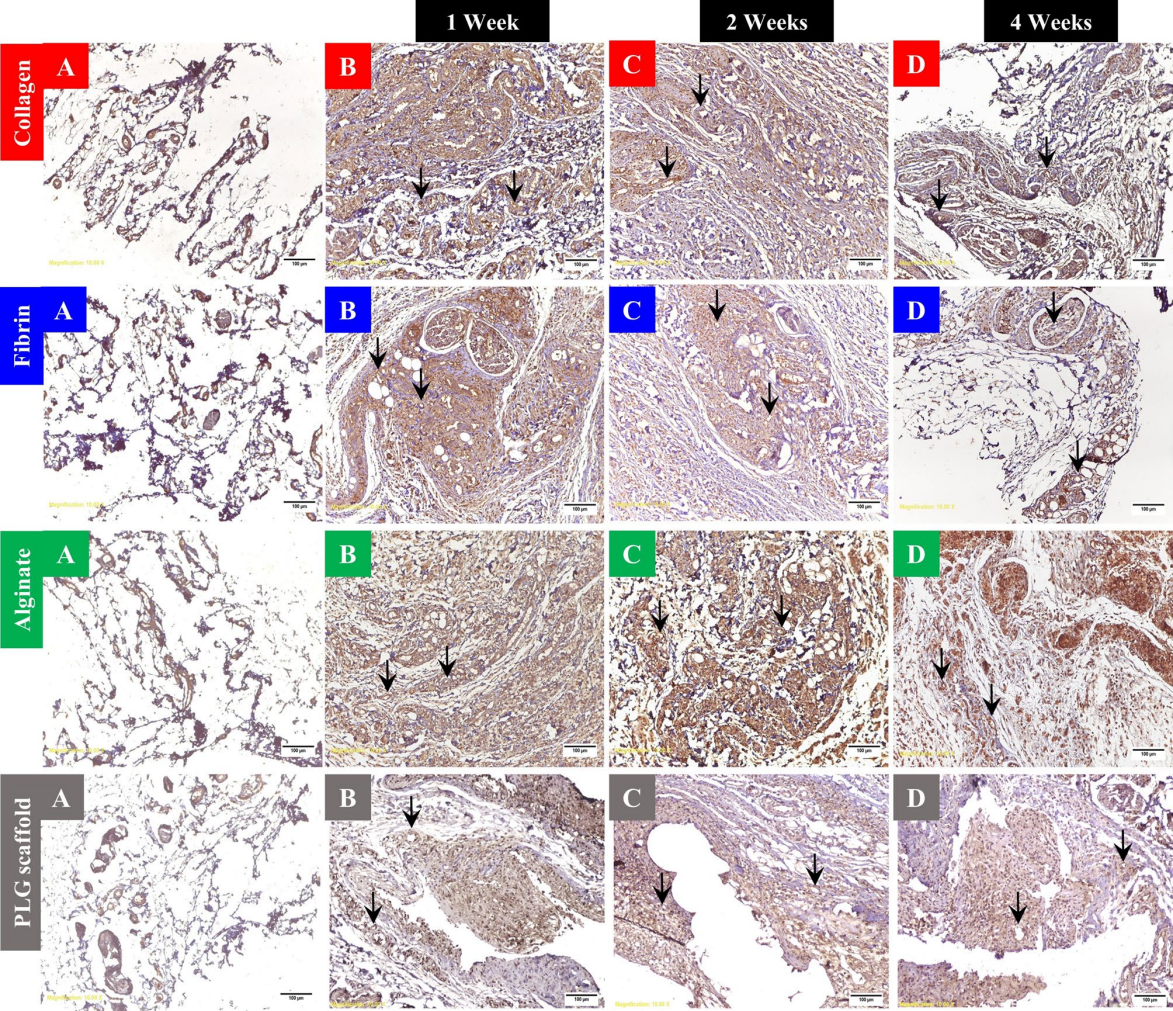
CD34 immunohistochemical staining of the subcutaneous tissues in all groups; × 10 magnification: **A**: Normal control tissue (baseline vascularity). **B**: One-week post-injection; there is a significant increase in CD34-positive vessels in the subcutaneous tissues treated with human collagen type I, human fibrin and alginate hydrogels. However, the increase in CD34-positive vessels was insignificant in the subcutaneous tissue implanted with PLG scaffold. Both alginate and PLG scaffold groups showed intense inflammatory infiltrates, which partially interfered with vessel quantification. **C**: Two weeks post-injection; CD34-positive vessels remained significantly elevated in the subcutaneous tissues treated by human collagen type I, human fibrin and alginate hydrogels. PLG scaffold-implanted tissues showed non significance increase in CD34-positive vessels. Inflammatory infiltrates persisted in both alginate and PLG groups. **D**: Four weeks post-injection; Vascular density remained significantly higher than control in human collagen type I, human fibrin and alginate groups. On the other hand, PLG scaffold-implanted tissues exhibited reduced vascular density. Yet, alginate and PLG scaffold groups quantification remained less reliable due to the persistent inflammation. CD34-positive cells appear brown. Black arrows indicate blood vessels. Scale bar = 100 μm.

In the human collagen type I group, immunohistochemical staining using CD34 antibody revealed blood vessel walls stained brown. CD34-positive blood vessels increased by 2.79-fold at week 1, 2.4-fold at week 2, and 1.94-fold at week 4, relative to the control tissues. These changes were statistically significant at all time points; *P* < 0.001 (Supplementary Fig. S1A and Supplementary Table S1). In the human fibrin group, CD34 immunohistochemistry revealed increased vascularity by 3.18-fold versus controls at week 1, 2.35-fold at week 2, and 2.3-fold at week 4. The progressive enhancement was statistically significant at all time points; *P* < 0.001 (Supplementary Fig. S1B and Supplementary Table S2). In the alginate group, quantification by CD34 immunohistochemistry was feasible and showed a marked angiogenic response. CD34-positive vessel counts increased by 3.96-fold at week 1 and by 3.53-fold at week 2. By week 4, vessel density declined to a 1.72-fold increase relative to baseline, though vascularity remained increased. These differences were statistically significant; *P* < 0.001 (Supplementary Fig. S1C and Supplementary Table S3). Nonspecific staining of inflammatory cells was noted but excluded from analysis. One animal in this group died due to an iatrogenic anesthetic complication. In the PLG scaffold group, CD34-positive vessels increased by 1.95-fold at week 1 and by 1.76-fold at week 2, followed by a reduction to a 1.22-fold increase at week 4. Despite this upward trend during the early phase, differences across time points were not statistically significant; *P* = 0.14 (Supplementary Fig. S1D and Supplementary Table S4). CD34 immunolabeling overlapped with inflammatory infiltrates in some regions; these areas were carefully excluded from quantitative analysis to ensure specificity (Fig. 3).

### Immunofluorescence (IF) staining

**Fig. 4:**
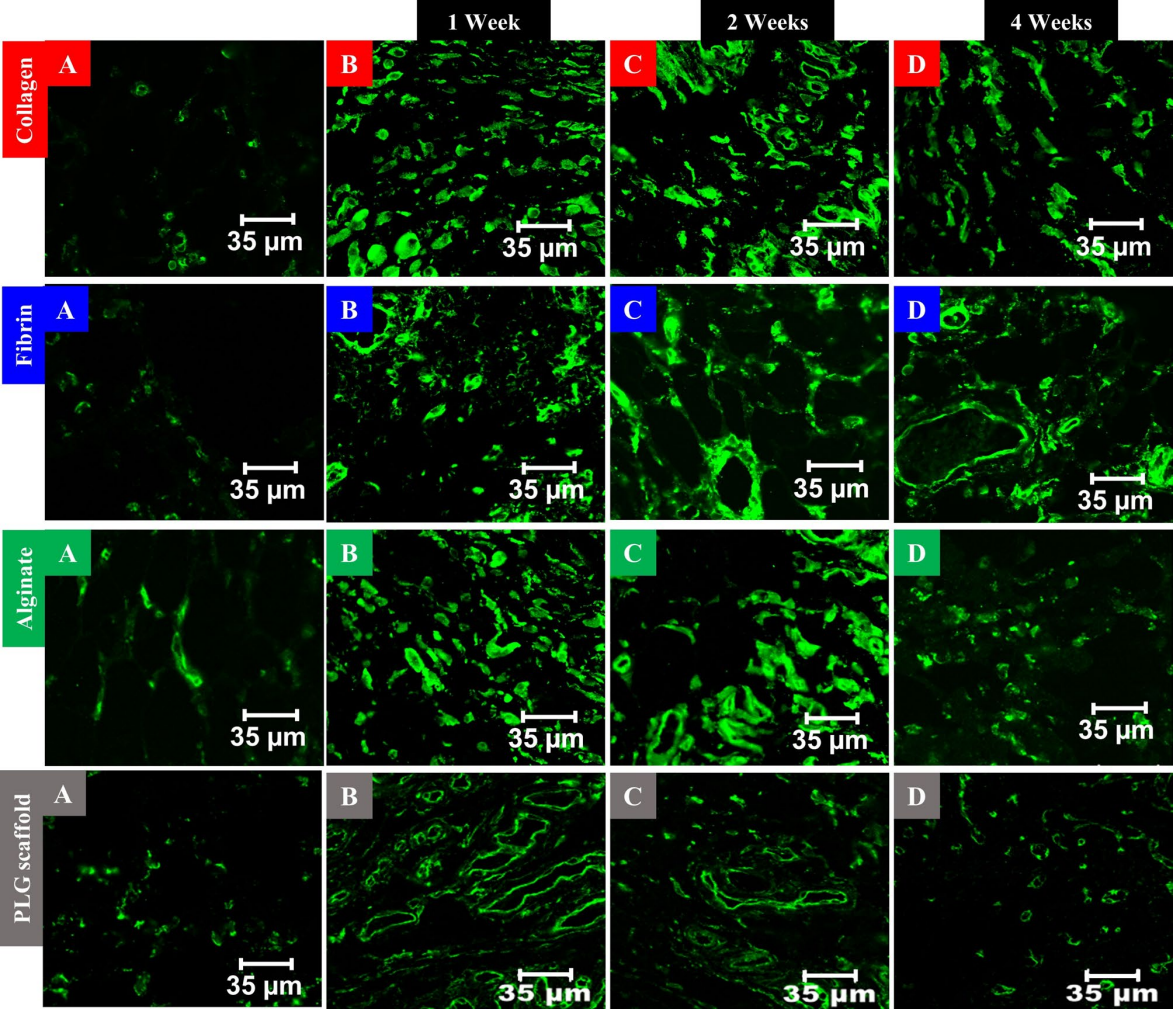
CD34 immunofluorescence staining of the subcutaneous tissues in all groups; ×60 magnification: **A**: Normal control group showed sparse CD34-positive staining, reflecting baseline vascularity in untreated tissue. **B**: One-week post-injection; human collagen type I and fibrin groups showed increased CD34-positive microvessels with early capillary-like structures. Alginate exhibited dense, disorganized vascular fluorescence with cellular infiltrates. PLG scaffold showed patchy CD34 expression within inflamed regions. **C**: Two weeks post-injection; human collagen type I and fibrin maintained enhanced, organized neovascular structures. Alginate remained highly vascularized but diffusely distributed amid inflammation. PLG scaffold showed moderate staining with persistent inflammatory zones. **D**: Four weeks post-injection; human collagen type I and fibrin sustained organized vascular networks but were slightly reduced compared to the previous time points. Alginate and PLG scaffold showed reduced CD34 signal and disrupted vessel patterns with ongoing inflammation. CD34-positive cells appeared green. Scale bar = 35 μm.

Consistent with the IHC findings, CD34 immunofluorescence staining in the human collagen type I and fibrin groups revealed well-formed capillary-like structures that became progressively more mature and spatially integrated over time. In the alginate group, immunofluorescence staining revealed intense but disorganized CD34-positive signals, especially at early time points, often associated with regions of dense inflammation. Vascular structures appeared less cohesive compared to the human collagen type I and fibrin groups. In the PLG scaffold group, CD34 immunofluorescence revealed patchy vascular staining and poorly formed vessel architecture associated with an intense inflammatory reaction (Fig. 4).

### Comparative analysis among all groups

**Fig. 5:**
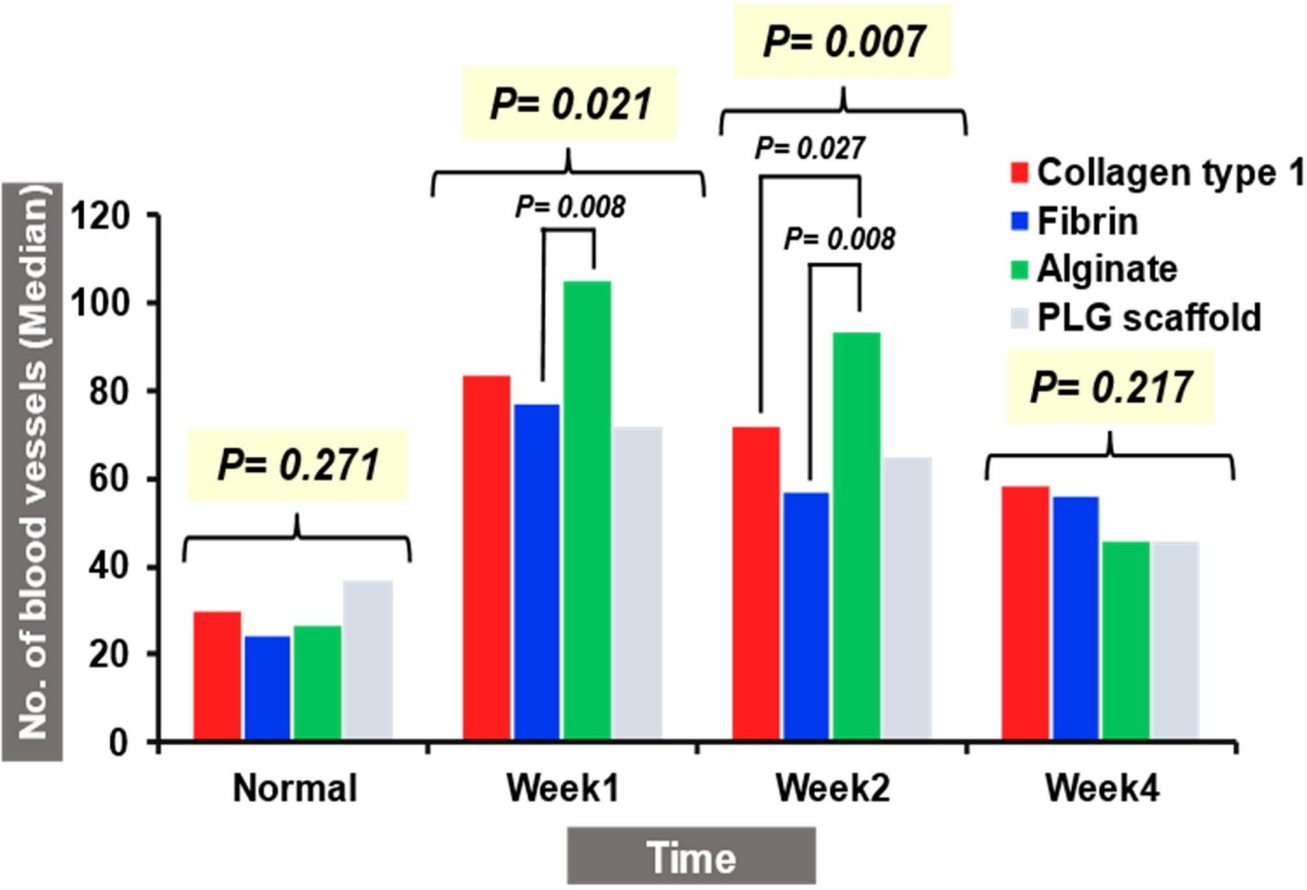
Comparative analysis of CD34⁺ vascular density in subcutaneous tissues treated with different biomaterials. Comparison of CD34⁺ vessel densities among all groups at each time point. At week 1, alginate showed the highest vascular density, with a significantly greater median vessel count than human fibrin (*P* = 0.021 overall). The neovascularization induced by human collagen type I, fibrin and PLG scaffold was comparable. At week 2, vessel density remained significantly different among groups (*P* = 0.007), with alginate again significantly higher than both human collagen type I (*P* = 0.027) and fibrin (*P* = 0.008). By week 4, no significant differences were observed among the materials (*P* = 0.217), although human collagen type I and fibrin retained slightly higher vessel counts than alginate and PLG scaffold.

The four biomaterials demonstrated enhanced neovascularization compared to baseline, with varying degrees of angiogenic response over time. At week 1, vessel density increased significantly across all groups (*P* = 0.021). Although human collagen type I (median: 83.75) and fibrin (median: 77.25) induced comparable levels of vascularization, no statistically significant differences were observed between them (*P* = 0.092). Alginate-treated tissues exhibited the highest vessel count (median: 105), significantly exceeding human fibrin (*P* = 0.008) but not human collagen type I (*P* = 0.417). PLG scaffold-treated tissues also showed an increase (median: 72), but this was not statistically different from any other groups (*P* > 0.05). At week 2, the inter-group difference remained significant (*P* = 0.007). Alginate maintained the highest median vessel count (93.5), significantly greater than both human collagen type I (*P* = 0.027) and fibrin (*P* = 0.008). Human collagen type I and fibrin showed similar vascular responses (medians: 72 and 57, respectively), with no significant difference (*P* = 0.059). The PLG scaffold group exhibited a modest increase (median: 65) but was not significantly different from any other groups (*P* > 0.05). By week 4, vessel density declined in all groups, and no statistically significant differences were detected among them (*P* = 0.217). Human collagen type I and fibrin maintained higher median values (58.25 and 56.25, respectively), while alginate (45.5) and PLG scaffold (45.5) showed greater reductions. Overall, while human collagen type I and fibrin hydrogels induced comparable neovascularization, alginate demonstrated significantly superior angiogenic effects at both week 1 and week 2. PLG scaffold showed modest, non-significant increases in vascular density (Supplementary Tables S5 and S6) (Fig. 5).

### Evaluation of inflammatory response and fibrosis

**Fig. 6:**
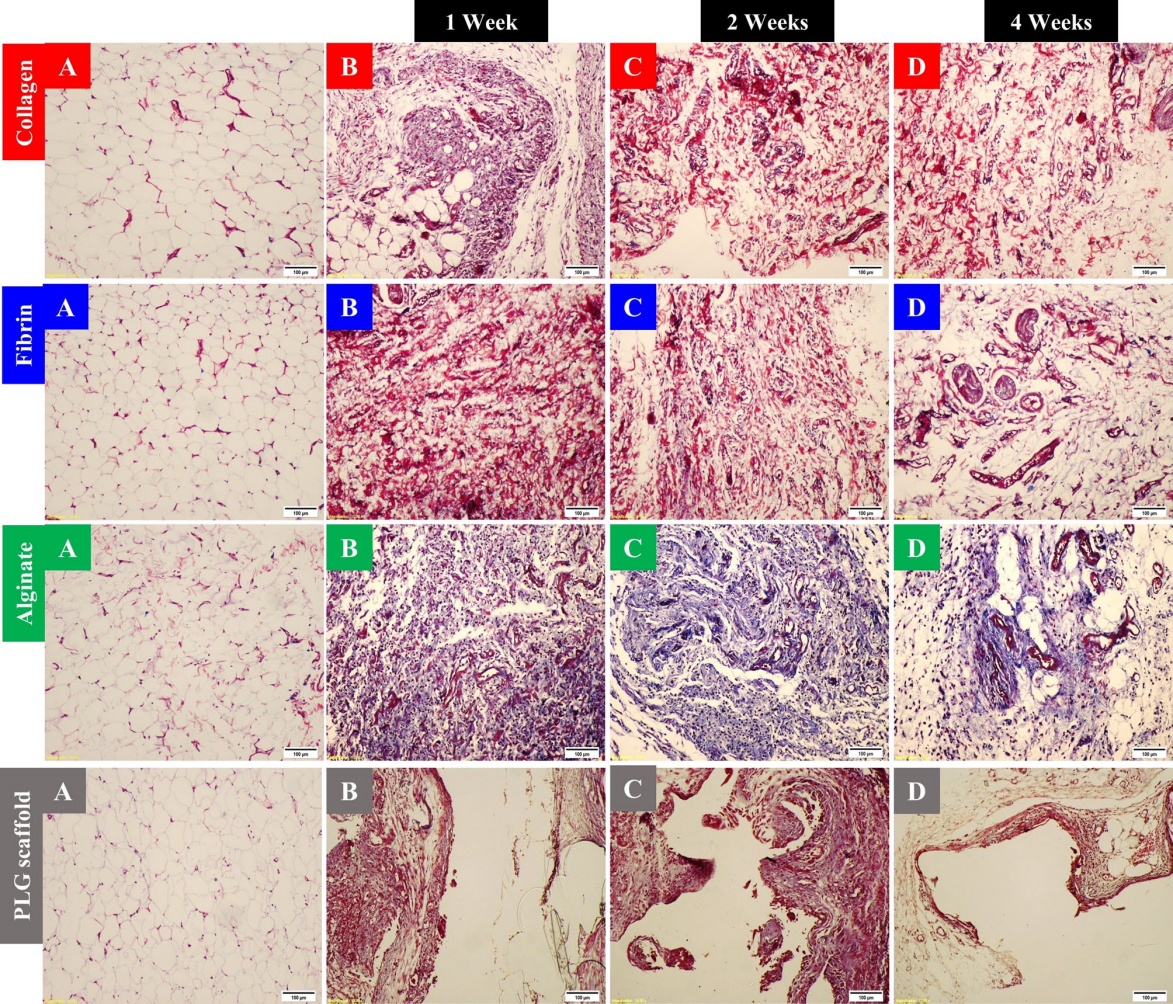
Histopathological examination of subcutaneous tissues for fibrosis in all groups (Masson’s Trichrome; × 10 magnification): **A**: Normal control tissue showed no fibrotic deposition, with clear adipose morphology. **B**: One-week post-injection; human collagen type I and fibrin groups exhibited no evidence of fibrosis. In contrast, alginate-treated tissues and PLG scaffold-implanted tissues displayed focal, blue-stained fibrotic areas. **C**: Two weeks post-injection; human collagen type I and fibrin groups remained fibrosis-free. Alginate and PLG scaffold groups induced fibrosis persistence, suggesting a sustained foreign body response. **D**: Four weeks post-injection; human collagen type I and fibrin treated tissues continued to show no fibrotic changes, while fibrosis was still detectable in alginate-treated rats. The PLG scaffold group demonstrated dense fibrotic changes, confirming its role as a positive control. Scale bar = 100 μm.

By H&E, both human collagen type I and fibrin -treated tissues showed no inflammatory infiltrates or fibrosis at any time point (Fig. 2). The negative Masson’s trichrome staining confirmed the absence of fibrotic deposition (Fig. 6).

In contrast, tissues injected with alginate displayed variable responses. H&E stain revealed a foreign body reaction, including the presence of epithelioid cells, multinucleated giant cells, and lymphocytes (Fig. 2). Likewise, Masson’s trichrome documented the development of fibrosis which stained blue (Fig. 6). The inflammatory tissue response was most prominent at week 1. At week 2, the inflammation had decreased and at week 4, only a minority of animals maintained inflammation and fibrosis.

Tissues implanted with PLG scaffolds, showed consistent foreign-body reaction (Fig. 2), including fibrous tissue formation (Fig. 6) and inflammatory cell infiltrates at all time points. Control tissues remained histologically normal.

## Discussion

The subcutaneous space represents a promising site for cell transplantation due to its easy accessibility and large cell volume capacity. It also offers advantages for biopsy, imaging, and retrieval possibilities. However, insufficient vascularity and the subsequent resulting hypoxia are considered major obstacles. Consequently, the subcutaneous environment fails to provide adequate oxygen and nutrients necessary for the viability of the transplanted cells. Several attempts at subcutaneous transplants have failed to achieve a successful cell engraftment owing to these fundamental constraints^39–41^. This underscores the urgent need for a strategy that induces robust subcutaneous neovascularization without eliciting a foreign body reaction^12^.

The two primary mechanisms of blood vessel formation are vasculogenesis and angiogenesis. Vasculogenesis is the *de novo* vessel formation from the coalescence of cells of the mesodermal lineage into tubular structures and is a hallmark of tissue development. Angiogenesis is the term used to describe the formation of new vessels from the already-existing vasculature via a process of sprouting and elongation, or via intussusception of the lumen. These processes are associated with tissue remodeling and repair. Yet, unregulated angiogenesis is a defining feature of cancer and other pathological conditions^42^. Therapeutic angiogenesis, the deliberate induction of neovessel formation, has demonstrated substantial therapeutic potential for treating different types of ischemic complications, enhancing wound healing, and supporting bone regeneration and tissue engineering^26^.

To overcome hypoxic and nutrient-deficient conditions in the subcutaneous space, both in vitro and in vivo prevascularization techniques have been developed to build a vascular network. An in vitro prevascularization strategy involves the culturing of vessel-forming cells, such as endothelial or endothelial progenitor cells on scaffolds before implantation. This approach promotes microvascular network formation, facilitating integration with host blood vessels post-implantation^25,26,43^. Conversely, in situ prevascularization harnesses the body to serve as a natural vasculature bioreactor. The reported angiogenic ingrowth technique requires surgical scaffold implantation into highly vascularized tissue, such as subcutaneous pockets or muscle. Host-derived blood vessels subsequently permeate and penetrate the construct over time^25,44,45^. Pepper et al. proposed a two-step approach in which plastic tubes were first implanted subcutaneously to promote angiogenesis, followed by cell transplantation into the prevascularized site^46^. Although successful, the multiple surgical procedures utilized limit their clinical applicability.

Biological hydrogel materials have emerged as important tools in vascular engineering and angiogenesis. They possess critical properties such as viscoelasticity and high-water content, closely mimicking the key features of the natural extracellular matrix (ECM)^47,48^. Notably, reports that employ only injectable biocompatible biological hydrogels for the creation of vascular bedding are scarce. In the current study, three biodegradable hydrogels were utilized for this purpose. The vascularization potential and tissue reaction of the three hydrogels were examined in the subcutaneous tissues of SD rats. Each rat served as its own control through sequential subcutaneous injections of the same biomaterial in different sites at certain time points, enabling the collection of both control and treated tissues at different time points from a single animal. Consequently, the study design minimized the undesired biological variability since rats exhibited distinct basal vascularity levels necessary for relevant biological assessments.

To ensure robust analysis, multiple histological techniques were employed to assess vascularization and tissue response. Neovascularization was assessed using the standard H&E staining, and CD34 IHC and IF to provide specific quantification and identification of the newly formed blood vessels. Additionally, fibrosis was assessed using Masson’s trichrome stain.

We evaluated the efficacy of various hydrogels injected at different time points, 1 week, 2 weeks, and 4 weeks. The tested materials included human-derived collagen type I and fibrin, as well as alginate, a biological material of non-human origin, in an attempt to advance their clinical application. Quantitative analysis revealed nearly *a* 3-fold increase in vascular density in the subcutaneous tissues treated with human collagen type I and fibrin hydrogels one week post injection. The vascular density gradually declined but remained significantly increased throughout the four weeks compared to the non-treated control quadrant. In spite of the higher vascular densities achieved by human collagen type I, human fibrin offers significant benefits of being inexpensive, fully autologous, and easily prepared from patient‑derived plasma^49^. As vascular density significantly increased after one week, it could be more appropriate to transplant cells one-week post injection of either human collagen type I or fibrin, coinciding with the peak vascular density.

Fortunately, human collagen type I and fibrin materials did not evoke foreign body reactions or fibrosis during the 4-week experimental period, ensuring their clinical biocompatibility. Importantly, while collagen is widely regarded as biocompatible, some collagen-based biomaterials, such as non-crosslinked porcine-derived membranes, have been shown to induce multinucleated giant cell formation and foreign body reactions in subcutaneous rat models^50^. This emphasizes the importance of the biological origin, crosslinking status, and processing of collagen as crucial factors which could affect its immunogenicity. The absence of such a response in this study highlights the favorable biocompatibility of the human collagen type I hydrogel used. Similarly, fibrin has been reported to induce foreign body responses and fibrosis. Balabiyev et al. demonstrated that fibrin polymer deposited on implant surfaces promotes macrophage fusion, foreign body giant cell formation, and the development of a dense collagen-rich fibrous capsule^51^. In contrast, the injectable human-derived fibrin hydrogel used in our study did not induce fibrosis or inflammatory reactions. These findings suggest that fibrin’s tissue response may be influenced by its physical form, structural presentation, and degradation kinetics. While alginate showed superior angiogenic activity at week 1 (3.96-fold), it was accompanied by dense inflammatory infiltrates and significant fibrosis by week 4. These undesired tissue responses hindered its clinical suitability despite its early vascular potential.

The assessment of the foreign body reaction is critical, as it can significantly influence constructs incorporating proteins, cells, or other biological components for regenerative applications^52^. Our study was controlled by implanting a PLG scaffold in the subcutaneous space, which is well-known to induce foreign body reactions^53^. Consistent with previous reports, the PLG scaffold elicited significant fibrosis and inflammatory reactions, in contrast to the inert response observed with human collagen type I and fibrin.

Despite the rapid and significant increase in blood vessel formation within the first week, it was followed by a gradual decrease in vascular density over time. Yet it remained significantly higher than the untreated control. This decline could be due to the lack of physiological demand in the injected area. This assumption aligned with the findings reported by DiPietro, who showed that after wound healing, the newly formed blood vessels stimulated by injury begin to regress^54^. Similarly, Krock et al. reported the effect of hypoxic conditions resulting from cellular metabolism on promoting vessel growth by upregulating pro-angiogenic pathways^55^. Subsequently, it would be beneficial to investigate whether physiological demand via the injection of cells into the hydrogel-treated area could potentially prevent vascular pruning.

Angiogenesis and vasculogenesis can be induced by mechanical cues^56^. The angiogenic effect of the three studied biomaterials may not be due to their biological structure and concentration only, but also due to their mechanical properties. Accordingly, the assessment of flow and deformation characteristics of the hydrogels by rheometer would provide a better understanding of their mechanical properties^57^. Future investigations of the signaling cascade pathways activated in response to blood vessel formation would clarify the mechanism of action of these hydrogels^58^. While inflammatory and fibrotic responses were assessed, the underlying immunological mechanisms warrant further investigation, as immune signaling pathways could influence cell graft integration and biocompatibility^59^.

The findings of the current study highlighted the dual potential of this approach towards achieving efficient biocompatible vascular network formation and opening new avenues for successful cell transplantation. The developed approach could establish crucial progress for clinical applications of tissue engineering and cellular therapy that combines the clinical utility and efficacy of a minimally invasive approach. It is worth mentioning that the approach used in this study was minimally invasive injections, thereby providing minimal intervention and minimizing the patient burden.

In spite of the promising findings reported in this study, some limitations should be considered. A longer follow-up would indicate whether newly formed blood vessels persist and grow or may decline over time. A larger sample size would strengthen the statistical power while ensuring replicable findings. Also, prior to the clinical application of this strategy, testing in a larger animal model is crucial. The assessment of vascularization could be further validated through additional histopathological evaluation by CD31^60,61^ and alpha-smooth muscle actin (α-SMA)^61,62^ markers. Unfortunately, the assessment of the perfusion capability of the newly formed blood vessels was not carried out in this experiment which require further studies to examine this crucial issue^63,64^.

## Conclusion

This study demonstrated that both human-derived biomaterials maintained biocompatibility and sustained vascular density over a four-week period, with peak effects observed at one week post-injection. On the other hand, alginate triggered intense inflammation and fibrosis that could limit its clinical applicability. These findings could support the use of injectable human collagen type I and fibrin as a promising, minimally invasive approach for the possible creation of a rich subcutaneous vascular network essential for the viability of transplanted cells.

## Supplementary Information

Below is the link to the electronic supplementary material.

Supplementary Material 1

## Data Availability

The datasets generated during and/or analyzed during the current study are available from the corresponding author on reasonable request.
