# Supplementary material for: Creation of a rich vascular subcutaneous space for cell transplantation via injectable biological hydrogels
Source: Sci Rep. 2025 Dec 20;15:44224. doi: 10.1038/s41598-025-29873-8 (PMC12722709; doi:10.1038/s41598-025-29873-8)
Supplement: Supplementary file 1 — Supplementary Material 1 [file 41598_2025_29873_MOESM1_ESM.docx]

***
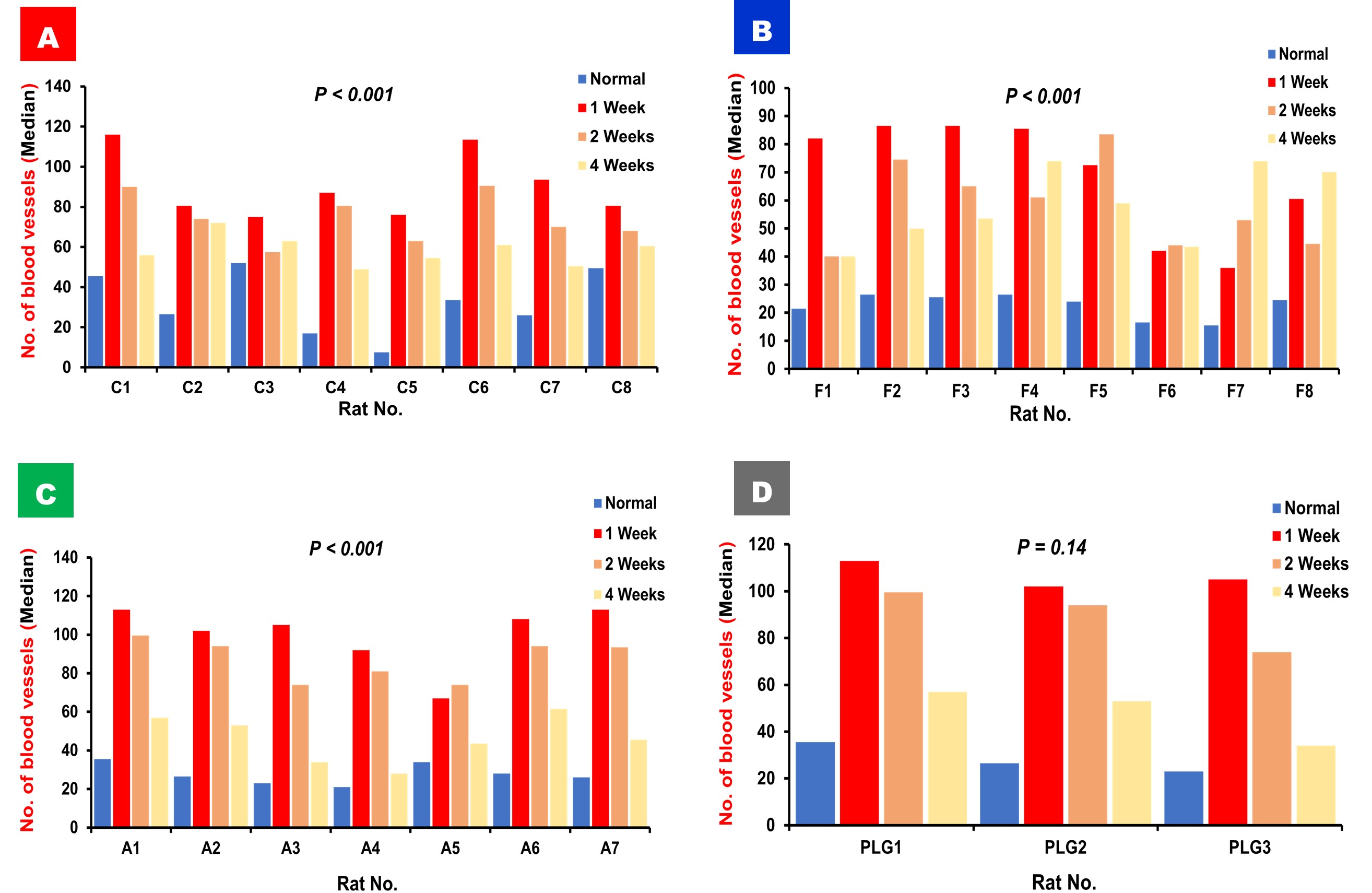
*Supplementary *Figure* S1:**

**Quantification of CD34⁺ vascular density in subcutaneous tissues treated with different biomaterials.**

1. Human collagen type I-treated tissues showed significant increases in CD34⁺ vessel density at all time points (*P < 0.001*), with a peak fold-increase at week 1 (2.79).
2. Human fibrin-treated tissues also exhibited significant neovascularization at all time points (*P < 0.001*), peaking at week 1 (3.18).
3. Alginate-treated tissues demonstrated the greatest vascular density increase (*P < 0.001*), with a peak fold-increase of 3.96 at week 1.
4. PLG scaffold-treated tissues showed non-significant increases in vessel density across all time points (*P = 0.14*), with a fold-increase of 1.95 at week 1.

**Supplementary Table S1:**

**Histopathological quantitation of blood vessels in the human collagen type I group at different time points:**

| Rat No. | Normal  median (Min, Max) | 1 Week  median (Min, Max) | 2 Weeks  median (Min, Max) | 4 Weeks  median (Min, Max) |  |
| --- | --- | --- | --- | --- | --- |
| **C1** | 45.5 (38, 55) | 116.5 (82,182) | 90 (46, 116) | 56 (45, 85) |  |
| **C2** | 26.5 (15, 35) | 80.5 (55, 88) | 74 (51, 92) | 72 (40, 92) |  |
| **C3** | 52 (20, 72) | 75.5 (50, 108) | 57.5 (37, 92) | 63 (40, 92) |  |
| **C4** | 17.5 (9, 31) | 87 (79, 93) | 80.5 (62, 88) | 49 (40, 66) |  |
| **C5** | 7.5 (6, 10) | 76 (49, 90) | 63 (40, 78) | 54.5 (30, 61) |  |
| **C6** | 33.5 (28, 43) | 113.5 (80, 164) | 90.5 (70, 124) | 61 (47, 82) |  |
| **C7** | 26 (21, 32) | 93.5 (79, 100) | 70 (40, 81) | 50.5 (40, 60) |  |
| **C8** | 49.5 (31, 60) | 80.5 (68, 88) | 68 (60, 80) | 60.5 (55, 66) |  |
| **Median (**Min, Max**) of collagen type I group** | 30 (7.5, 52) | 83.75 (75, 116) | 72 (57.5, 90.5) | 58.25 (49, 72) | ***P˂ 0.001*** |

**Supplementary Table S2:**

**Histopathological quantitation of blood vessels in the human fibrin group at different time points:**

| Rat No. | Normal  median (Min, Max) | 1 Week  median (Min, Max) | 2 Weeks  median (Min, Max) | 4 Weeks  median (Min, Max) |  |
| --- | --- | --- | --- | --- | --- |
| **F1** | 21.5 (19, 26) | 82 (38,108) | 40 (28,49) | 40 (20, 49) |  |
| **F2** | 26.5 (20, 31) | 86.5 (51, 128) | 74.5 (53, 92) | 50 (40, 69) |  |
| **F3** | 25.5 (20, 30) | 86.5 (70, 126) | 65 (49, 93) | 53.5 (28, 71) |  |
| **F4** | 26.5 (21, 31) | 85.5 (50, 148) | 61 (38, 122) | 74 (49, 106) |  |
| **F5** | 24 (20, 29) | 72.5 (45, 113) | 83.5 (50, 100) | 59 (20, 74) |  |
| **F6** | 16.5 (9, 39) | 42 (21, 58) | 44 (25, 66) | 43.5 (25, 63) |  |
| **F7** | 15.5 (11, 20) | 36 (30, 41) | 53 (40, 68) | 74.5 (70, 79) |  |
| **F8** | 24.5 (20, 29) | 60.5 (21, 80) | 44.5 (33, 90) | 70 (66, 83) |  |
| **Median (**Min, Max**) of fibrin group** | 24.25 (15.5, 26.5) | 77.25 (36, 86.5) | 57.00 (40, 83.5) | 56.25 (40, 74) | ***P˂ 0.001*** |

**Supplementary Table S3:**

**Histopathological quantitation of blood vessels in the alginate group at different time points:**

| Rat No. | Normal  median (Min, Max) | 1 Week  median (Min, Max) | 2 Weeks  median (Min, Max) | 4 Weeks  median (Min, Max) |  |
| --- | --- | --- | --- | --- | --- |
| **A1** | 35.5 (14, 60) | 113 (77, 166) | 99.5 (58, 146) | 57 (33, 72) |  |
| **A2** | 26.5 (12, 44) | 102 (76, 162) | 94 (68, 124) | 53 (28, 90) |  |
| **A3** | 23 (16, 33) | 105 (74, 142) | 74 (55, 104) | 34 (18, 44) |  |
| **A4** | 21 (12, 46) | 92 (64, 114) | 81 (62, 118) | 28 (18, 40) |  |
| **A5** | 34 (22, 68) | 67 (52, 82) | 74 (54, 98) | 43.5 (24, 66) |  |
| **A6** | 28 (18, 48) | 108 (86, 168) | 94 (68, 124) | 61.5 (46, 96) |  |
| **A7** | 26 (16, 36) | 113 (86, 182) | 93.5 (66, 122) | 45.5 (34, 52) |  |
| **Median (**Min, Max**) of alginate group** | 26.5 (21, 35.5) | 105 (67, 113) | 93.5 (74, 99.5) | 45.5 (28, 61.5) | ***P˂ 0.001*** |

**Supplementary Table S4:**

**Histopathological quantitation of blood vessels in the PLG scaffold group at different time points:**

| Rat No. | Normal  median (Min, Max) | 1 Week  median (Min, Max) | 2 Weeks  median (Min, Max) | 4 Weeks  median (Min, Max) |  |
| --- | --- | --- | --- | --- | --- |
| **PLG1** | 39 (24, 58) | 72 (52, 102) | 88 (68, 122) | 50 (32, 72) |  |
| **PLG2** | 37 (24, 66) | 71 (52, 98) | 33 (22, 64) | 26.5 (18, 40) |  |
| **PLG3** | 27 (18, 42) | 79.5 (54, 104) | 65 (48, 82) | 45.5 (34, 60) |  |
| **Median (**Min, Max**) of PLG scaffold group** | 37 (27, 39) | 72 (71, 79) | 65 (33, 88) | 45.5 (26, 50) | ***P = 0.14*** |

**Supplementary Table S5:**

**Comparison of blood vessel numbers among all groups at different time points:**

| Material | Normal  median (Min, Max) | 1 Week  median (Min, Max) | 2 Weeks  median (Min, Max) | 4 Weeks  median (Min, Max) | *P* Value |
| --- | --- | --- | --- | --- | --- |
| **Collagen type I** | 30 (7.5, 52) | 83.75 (75, 116) | 72 (57.5, 90.5) | 58.25 (49, 72) | ***< 0.001*** |
| **Fibrin** | 24.25 (15.5, 26.5) | 77.25 (36, 86.5) | 57 (40, 83.5) | 56.25 (40, 74) | ***< 0.001*** |
| **Alginate** | 26.5 (21, 35.5) | 105 (67, 113) | 93.5 (74, 99.5) | 45.5 (28, 61.5) | ***< 0.001*** |
| **PLG scaffold** | 37 (27, 39) | 72 (71, 79) | 65 (33, 88) | 45.5 (26, 50) | ***= 0.14*** |
| ***P* Value** | ***0.271*** | ***0.021*** | ***0.007*** | ***0.217*** | - |

**Supplementary Table S6:**

**Pairwise comparison of blood vessel numbers among groups at weeks 1 (W1) and 2 (W2):**

| **Material** | **Collagen type I** | **Fibrin** | **Alginate** |
| --- | --- | --- | --- |
| **Collagen type I** | - | - | - |
| **Fibrin** | **W1** **(***P= 0.092***),**  **W2 (***P= 0.059***)** | - | - |
| **Alginate** | **W1 (***P= 0.417***),**  **W2 (***P= 0.027***)** | **W1 (***P= 0.008***),**  **W2 (***P= 0.008***)** | - |
| **PLG scaffold** | **W1 (***P= 0.22***),**  **W2 (***P= 0.082***)** | **W1 (***P= 0.14***),**  **W2 (***P= 0.41***)** | **W1 (***P= 0.728***),**  **W2 (***P= 0.639***)** |
